# (η^6^-Benzophenone)(η^5^-penta­methyl­cyclo­penta­dien­yl)ruthenium(II) tetra­phenyl­borate

**DOI:** 10.1107/S1600536809042731

**Published:** 2009-10-31

**Authors:** Bradley T. Loughrey, Kevin Nadin, Michael L. Williams, Peter C. Healy

**Affiliations:** a Eskitis Institute for Cell and Molecular Therapies, Griffith University, Brisbane 4111, Australia; b Département de Chimie, Faculté des Sciences, Université d’Orléans, Orléans BP 6759 45067, France

## Abstract

The structure of the title compound, [Ru(C_10_H_15_)(C_13_H_10_O)](C_24_H_20_B), consists of discrete [Cp*Ru(II)benzophenone] cations and tetra­phenyl­borate anions (Cp* = penta­methyl­cyclo­penta­dien­yl). Tethering the Cp*Ru group to one aryl ring of benzophenone results in average values of 1.42 (1) and 1.38 (1) Å for the C—C bond lengths in the Ru-tethered and untethered phenyl rings, respectively. The dihedral angle between the benzene and phenyl rings of the benzophenone group is 50.5 (1)°.

## Related literature

For background to our research into the structural and biological properties of ionic Ru(II) organometallic complex salts [Cp*Ru(II)-arene]^+^ *X* ^−^, see: Loughrey *et al.* (2008 ▶, 2009 ▶); For related structures, see: Moncol & Coppens (2004 ▶); Gemel *et al.* (1996 ▶).
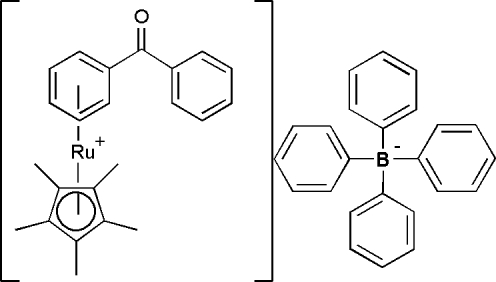


## Experimental

### 

#### Crystal data

[Ru(C_10_H_15_)(C_13_H_10_O)](C_24_H_20_B)
*M* *_r_* = 737.71
Monoclinic, 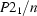

*a* = 11.6754 (8) Å
*b* = 20.5730 (12) Å
*c* = 15.6245 (9) Å
β = 91.250 (6)°
*V* = 3752.1 (4) Å^3^
*Z* = 4
Mo *K*α radiation
μ = 0.45 mm^−1^
*T* = 296 K
0.30 × 0.27 × 0.13 mm

#### Data collection

Oxford-Diffraction GEMINI S Ultra diffractometer
Absorption correction: multi-scan (*CrysAlis RED*; Oxford Diffraction, 2007 ▶) *T*_min_ = 0.876, *T*_max_ = 0.943
34346 measured reflections
8604 independent reflections
6521 reflections with *I* > 2σ(*I*)
*R*_int_ = 0.045

#### Refinement

*R*[*F* ^2^ > 2σ(*F* ^2^)] = 0.038
*wR*(*F* ^2^) = 0.110
*S* = 1.02
8604 reflections
451 parameters
H-atom parameters constrained
Δρ_max_ = 1.01 e Å^−3^
Δρ_min_ = −0.31 e Å^−3^

### 

Data collection: *CrysAlis CCD* (Oxford Diffraction, 2007 ▶); cell refinement: *CrysAlis RED* (Oxford Diffraction, 2007 ▶); data reduction: *CrysAlis RED*; program(s) used to solve structure: *SIR97* (Altomare *et al.*, 1999 ▶); program(s) used to refine structure: *SHELXL97* (Sheldrick, 2008 ▶); molecular graphics: *ORTEP-3 for Windows* (Farrugia, 1997 ▶); software used to prepare material for publication: *PLATON* (Spek, 2009 ▶).

## Supplementary Material

Crystal structure: contains datablocks global, I. DOI: 10.1107/S1600536809042731/lh2927sup1.cif

Structure factors: contains datablocks I. DOI: 10.1107/S1600536809042731/lh2927Isup2.hkl

Additional supplementary materials:  crystallographic information; 3D view; checkCIF report

## Figures and Tables

**Fig. 1.**
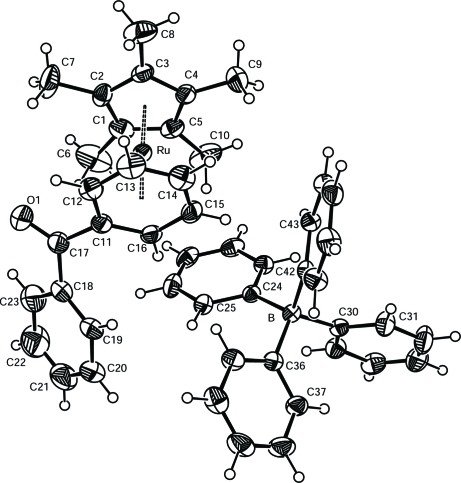
The asymmetric unit of (I), with atom labels and 40% probability displacement ellipsoids for the non-H atoms.

## supplementary crystallographic information

### Comment

As part of our continued investigation into the structural and biological properties of ionic Ru(II) organometallic complex salts [Cp*Ru(II)-arene]^+^X^-^ (Loughrey *et al.*,  2008, 2009), we have prepared the mono substituted adduct of Cp*Ru and benzophenone as the tetraphenylborate salt. The compound was prepared in 49% yield through the reaction of RuCl_3_.xH_2_O with an excess of benzophenone and HCp* in ethanol and isolated as the crystalline tetraphenylborate salt through addition of an aqueous solution of NaBPh_4_.

The compound crystallizes as discrete cations and anions (Fig. 1). No significant C-H···O interactions are observed between the phenyl and methyl protons and the ketone oxygen. The dihedral angle between the two aryl rings of the benzophenone is 50.5 (1)° and comparable to the value of 53.75 (6)° reported for the parent monoclinic benzophenone (Moncol & Coppens, 2004).  The Ru-C bond lengths in the cation are also similar to those reported for other [Cp*Ru(arene)]^+^ complexes (Gemel *et al.*,  1996; Loughrey *et al.*,  2008). The average value of 1.42 (1)Å for the C-C bond lengths in the Ru tethered phenyl ring (C11-C16) is 0.04Å longer than the average value of 1.38 (1)Å observed for the free phenyl ring (C18-C23), the latter being marginally shorter than the value of 1.392 (5)Å for the parent benzophenone.

### Experimental

Benzophenone (0.8 g, 4.39 mmol) and HCp* (0.3 ml, 1.88 mmol) were added to a solution of ruthenium trichloride hydrate (0.20 g, 0.76 mmol) in ethanol (20 ml) under argon. The resulting solution was heated under reflux conditions for a period of 10 h to yield a golden brown coloured solution. The solvent was concentrated *in vacuo* with the remaining residue being redissolved in an ether/water partition (20 ml/20 ml). The aqueous portion was retained and washed with a further three aliquots of diethyl ether (20 ml). The aqueous layer was mixed slowly with an aqueous solution of sodium tetraphenylborate (5 ml, 0.30 *M*). The resulting cream coloured precipitate was filtered from solution and redissolved in a minimum quantity of acetone. This solution was filtered through a short alumina column (neutral, 150 mesh) using acetone as the eluent. The solution was concentrated *in vacuo* and the product recrystallized through addition of a minimum quantity of cold water. The resulting crystalline precipitate was then filtered from solution and dried *in vacuo*. Yield = 0.273 g, 48.7%. Crystals suitable for X-ray diffraction studies were grown by slow diffusion of diethyl ether into a solution of the compound in acetone.

NMR: ^1^H (d_6_DMSO), δ 1.81 (s, 15H, C_5_(C_5_H_15_)), 6.07–6.11 (m, 3H, C_6_H_5_*meta* and *para*), 6.30–6.31 (m, 2H, C_6_H_5_*ortho*), 6.72–6.75 (m, 4H, B(C_6_H_5_)_4_*para*), 6.85–6.89 (m, 8H, B(C_6_H_5_)_4_*meta*), 7.15–7.18 (m, 8H, B(C_6_H_5_)_4_*ortho*), 7.54–7.7.58 (m, 2H, C_6_H_5_*meta*), 7.69–7.72 (m, 1H, C_6_H_5_*para*), 7.79–7.82 (m, 2H, C_6_H_5_*ortho*). ^13^C (d_6_DMSO), δ 9.74 (C5 (C_5_H_15_)), 87.42 (2CH, aromatic), 87.67 (2CH, aromatic), 89.03 (1CH, aromatic), 94.82 (1CH, aromatic), 97.06 (C_5_(C_5_H_15_)), 121.43 (4CH, B(C_6_H_5_)_4_), 125.22 (8CH, B(C_6_H_5_)_4_), 128.89 (2CH, aromatic), 129.48 (2CH, aromatic), 133.83 (1CH, aromatic), 135.48 (8CH, B(C_6_H_5_)_4_), 135.79 (1CH, aromatic), 162.72, 163.12, 163.51, 163.90 (4CH, B(C_6_H_5_)_4_), (signals split by ^11^B), 192.86 (C=O). ESMS (m/*z*) +ve ion, calcd m/*z* for [(η^5^-C_5_(CH_3_)_5_) Ru(η^6^-C_6_H_5_COC_6_H_5_)+] 418.51, found 418.85 (100%), -ve ion, calcd m/*z* for B(C_6_H_5_)_4_ 319.25, found 319.05 (100%). calcd % for C_47_H_45_OBRu C 76.5, H 6.15, found C 76.2, H 6.14.

### Refinement

H atoms attached to carbon were constrained as riding atoms, with C–H set to 0.94–96 Å. *U*_iso_(H) values were set to 1.2*U*_eq_ (aromatic) and 1.5*U*_eq_ (alkyl) of the parent atom.

### Crystal data

**Table:**

C_23_H_25_ORu^+^·C_24_H_20_B^−^	*F*(000) = 1536
*M**_r_* = 737.71	*D*_x_ = 1.306 Mg m^−^^3^
Monoclinic, *P*2_1_/*n*	Mo *K*α radiation, λ = 0.71070 Å
Hall symbol: -P 2yn	Cell parameters from 10435 reflections
*a* = 11.6754 (8) Å	θ = 2.8–32.8°
*b* = 20.5730 (12) Å	µ = 0.45 mm^−^^1^
*c* = 15.6245 (9) Å	*T* = 296 K
β = 91.250 (6)°	Prism, colourless
*V* = 3752.1 (4) Å^3^	0.30 × 0.27 × 0.13 mm
*Z* = 4	

### Data collection

**Table:**

Oxford-Diffraction GEMINI S Ultra diffractometer	8604 independent reflections
Radiation source: Enhance (Mo) X-ray Source	6521 reflections with *I* > 2σ(*I*)
graphite	*R*_int_ = 0.045
Detector resolution: 16.0774 pixels mm^-1^	θ_max_ = 27.5°, θ_min_ = 2.8°
ω and φ scans	*h* = −15→15
Absorption correction: multi-scan (*CrysAlis RED*; Oxford Diffraction, 2007)	*k* = −26→26
*T*_min_ = 0.876, *T*_max_ = 0.943	*l* = −20→20
34346 measured reflections	

### Refinement

**Table:**

Refinement on *F*^2^	Primary atom site location: structure-invariant direct methods
Least-squares matrix: full	Secondary atom site location: difference Fourier map
*R*[*F*^2^ > 2σ(*F*^2^)] = 0.038	Hydrogen site location: inferred from neighbouring sites
*wR*(*F*^2^) = 0.110	H-atom parameters constrained
*S* = 1.02	*w* = 1/[σ^2^(*F*_o_^2^) + (0.0688*P*)^2^] where *P* = (*F*_o_^2^ + 2*F*_c_^2^)/3
8604 reflections	(Δ/σ)_max_ = 0.001
451 parameters	Δρ_max_ = 1.01 e Å^−^^3^
0 restraints	Δρ_min_ = −0.31 e Å^−^^3^

### Special details

**Table:**

Experimental. Empirical absorption correction using spherical harmonics, implemented in SCALE3 ABSPACK scaling algorithm.
Geometry. Bond distances, angles *etc*. have been calculated using the rounded fractional coordinates. All su's are estimated from the variances of the (full) variance-covariance matrix. The cell e.s.d.'s are taken into account in the estimation of distances, angles and torsion angles
Refinement. Refinement on *F*^2^ for ALL reflections except those flagged by the user for potential systematic errors. Weighted *R*-factors *wR* and all goodnesses of fit *S* are based on *F*^2^, conventional *R*-factors *R* are based on *F*, with *F* set to zero for negative *F*^2^. The observed criterion of *F*^2^ > σ(*F*^2^) is used only for calculating -*R*-factor-obs *etc*. and is not relevant to the choice of reflections for refinement. *R*-factors based on *F*^2^ are statistically about twice as large as those based on *F*, and *R*-factors based on ALL data will be even larger.

### Fractional atomic coordinates and isotropic or equivalent isotropic displacement parameters (Å^2^)

**Table:**

	*x*	*y*	*z*	*U*_iso_*/*U*_eq_	
Ru	0.84997 (2)	−0.09802 (1)	0.20184 (1)	0.0324 (1)	
O1	1.08769 (19)	−0.03654 (13)	0.34508 (17)	0.0756 (10)	
C1	0.9535 (3)	−0.04791 (15)	0.10949 (17)	0.0482 (9)	
C2	0.9916 (2)	−0.11426 (15)	0.11576 (17)	0.0450 (9)	
C3	0.8987 (2)	−0.15534 (13)	0.09019 (17)	0.0421 (8)	
C4	0.8045 (2)	−0.11479 (15)	0.06727 (17)	0.0442 (9)	
C5	0.8379 (3)	−0.04852 (14)	0.07874 (17)	0.0456 (9)	
C6	1.0233 (4)	0.0116 (2)	0.1288 (3)	0.0896 (16)	
C7	1.1100 (3)	−0.1364 (3)	0.1403 (2)	0.0868 (16)	
C8	0.8996 (4)	−0.22797 (17)	0.0844 (3)	0.0762 (16)	
C9	0.6913 (3)	−0.1390 (2)	0.0327 (2)	0.0752 (14)	
C10	0.7649 (4)	0.01049 (19)	0.0591 (3)	0.0808 (14)	
C11	0.8908 (2)	−0.05747 (14)	0.33131 (16)	0.0405 (8)	
C12	0.9067 (3)	−0.12651 (15)	0.33336 (18)	0.0477 (9)	
C13	0.8181 (3)	−0.16832 (15)	0.3068 (2)	0.0545 (10)	
C14	0.7132 (3)	−0.14249 (16)	0.2773 (2)	0.0544 (10)	
C15	0.6951 (2)	−0.07452 (16)	0.27371 (18)	0.0482 (9)	
C16	0.7850 (2)	−0.03171 (13)	0.30091 (17)	0.0411 (8)	
C17	0.9926 (2)	−0.01548 (16)	0.35571 (18)	0.0499 (10)	
C18	0.9745 (3)	0.05050 (16)	0.39330 (19)	0.0515 (10)	
C19	0.8771 (3)	0.06664 (18)	0.43805 (19)	0.0562 (11)	
C20	0.8658 (3)	0.1280 (2)	0.4726 (2)	0.0742 (14)	
C21	0.9517 (4)	0.1732 (2)	0.4637 (3)	0.0858 (16)	
C22	1.0469 (4)	0.1575 (2)	0.4208 (4)	0.0972 (19)	
C23	1.0602 (3)	0.0966 (2)	0.3852 (3)	0.0757 (16)	
C24	0.5831 (2)	0.14110 (11)	0.20605 (16)	0.0324 (7)	
C25	0.6876 (2)	0.14672 (13)	0.25259 (18)	0.0401 (8)	
C26	0.7888 (2)	0.16572 (14)	0.2137 (2)	0.0514 (10)	
C27	0.7897 (2)	0.18102 (14)	0.1282 (2)	0.0503 (10)	
C28	0.6898 (3)	0.17746 (14)	0.08089 (19)	0.0471 (9)	
C29	0.5894 (2)	0.15813 (13)	0.11926 (17)	0.0386 (8)	
C30	0.3553 (2)	0.15999 (12)	0.20889 (15)	0.0352 (8)	
C31	0.2454 (2)	0.13611 (14)	0.1953 (2)	0.0510 (10)	
C32	0.1541 (3)	0.17538 (18)	0.1707 (3)	0.0676 (13)	
C33	0.1688 (3)	0.24065 (17)	0.1575 (2)	0.0642 (12)	
C34	0.2755 (3)	0.26668 (14)	0.1697 (2)	0.0554 (11)	
C35	0.3669 (2)	0.22688 (13)	0.19460 (19)	0.0446 (9)	
C36	0.4634 (2)	0.12033 (12)	0.35293 (16)	0.0343 (7)	
C37	0.3847 (2)	0.15666 (14)	0.39862 (18)	0.0461 (9)	
C38	0.3842 (3)	0.15708 (18)	0.4883 (2)	0.0631 (11)	
C39	0.4635 (3)	0.1221 (2)	0.53473 (19)	0.0657 (13)	
C40	0.5417 (3)	0.08492 (17)	0.4927 (2)	0.0562 (10)	
C41	0.5402 (2)	0.08402 (14)	0.40433 (18)	0.0446 (9)	
C42	0.43975 (19)	0.03810 (12)	0.22098 (16)	0.0323 (7)	
C43	0.4613 (2)	0.01326 (13)	0.13928 (17)	0.0405 (8)	
C44	0.4402 (2)	−0.05134 (15)	0.1166 (2)	0.0506 (10)	
C45	0.3970 (3)	−0.09398 (14)	0.1757 (2)	0.0560 (10)	
C46	0.3724 (3)	−0.07151 (16)	0.2559 (2)	0.0557 (10)	
C47	0.3925 (2)	−0.00675 (14)	0.27752 (19)	0.0468 (9)	
B	0.4619 (2)	0.11538 (13)	0.24744 (18)	0.0321 (8)	
H6A	0.98700	0.04960	0.10410	0.1040*	
H6B	1.03270	0.01840	0.18850	0.1040*	
H6C	1.09810	0.00830	0.10420	0.1040*	
H7A	1.11990	−0.13960	0.20060	0.1000*	
H7B	1.16660	−0.10750	0.11850	0.1000*	
H7C	1.12480	−0.17910	0.11620	0.1000*	
H8A	0.96550	−0.24330	0.05630	0.0890*	
H8B	0.83270	−0.24290	0.05240	0.0890*	
H8C	0.89710	−0.24700	0.14020	0.0890*	
H9A	0.64100	−0.14870	0.07890	0.0890*	
H9B	0.65450	−0.10670	−0.00270	0.0890*	
H9C	0.70060	−0.17750	−0.00030	0.0890*	
H10A	0.80590	0.04130	0.02630	0.1000*	
H10B	0.69730	−0.00220	0.02620	0.1000*	
H10C	0.74040	0.03050	0.11050	0.1000*	
H12	0.97920	−0.14420	0.35250	0.0580*	
H13	0.82930	−0.21460	0.30860	0.0650*	
H14	0.65290	−0.17200	0.25940	0.0670*	
H15	0.62300	−0.05710	0.25340	0.0580*	
H16	0.77370	0.01430	0.29840	0.0500*	
H19	0.81830	0.03540	0.44490	0.0680*	
H20	0.79890	0.13870	0.50360	0.0880*	
H21	0.94270	0.21560	0.48740	0.1040*	
H22	1.10700	0.18930	0.41450	0.1160*	
H23	1.12850	0.08600	0.35620	0.0910*	
H25	0.68910	0.13690	0.31130	0.0480*	
H26	0.85790	0.16800	0.24770	0.0610*	
H27	0.85940	0.19390	0.10250	0.0630*	
H28	0.68950	0.18850	0.02210	0.0570*	
H29	0.52110	0.15650	0.08510	0.0470*	
H31	0.23240	0.09060	0.20300	0.0620*	
H32	0.07990	0.15690	0.16170	0.0840*	
H33	0.10560	0.26750	0.14090	0.0770*	
H34	0.28790	0.31180	0.16050	0.0670*	
H35	0.44020	0.24600	0.20200	0.0530*	
H37	0.32830	0.18130	0.36800	0.0560*	
H38	0.32950	0.18220	0.51690	0.0770*	
H39	0.46360	0.12380	0.59520	0.0800*	
H40	0.59630	0.05990	0.52460	0.0680*	
H41	0.59420	0.05720	0.37610	0.0540*	
H43	0.49270	0.04110	0.09770	0.0470*	
H44	0.45330	−0.06550	0.06030	0.0590*	
H45	0.38460	−0.13820	0.16090	0.0680*	
H46	0.34270	−0.10060	0.29630	0.0690*	
H47	0.37360	0.00730	0.33310	0.0570*	

### Atomic displacement parameters (Å^2^)

**Table:**

	*U*^11^	*U*^22^	*U*^33^	*U*^12^	*U*^13^	*U*^23^
Ru	0.0338 (1)	0.0337 (1)	0.0298 (1)	0.0032 (1)	0.0029 (1)	0.0022 (1)
O1	0.0451 (13)	0.0924 (19)	0.0891 (18)	0.0093 (12)	−0.0040 (12)	−0.0270 (15)
C1	0.0593 (18)	0.0520 (17)	0.0337 (14)	−0.0123 (14)	0.0128 (12)	0.0010 (13)
C2	0.0361 (14)	0.0651 (19)	0.0339 (14)	0.0090 (13)	0.0056 (11)	0.0071 (13)
C3	0.0474 (15)	0.0443 (15)	0.0349 (14)	0.0137 (12)	0.0059 (11)	−0.0021 (12)
C4	0.0428 (15)	0.0570 (18)	0.0328 (14)	0.0118 (12)	−0.0013 (11)	−0.0044 (12)
C5	0.0566 (17)	0.0473 (16)	0.0333 (14)	0.0146 (13)	0.0086 (12)	0.0062 (12)
C6	0.119 (3)	0.085 (3)	0.066 (2)	−0.055 (3)	0.030 (2)	−0.005 (2)
C7	0.0366 (17)	0.160 (4)	0.064 (2)	0.026 (2)	0.0057 (15)	0.015 (3)
C8	0.105 (3)	0.049 (2)	0.075 (3)	0.0209 (19)	0.013 (2)	−0.0080 (18)
C9	0.0530 (19)	0.109 (3)	0.063 (2)	0.005 (2)	−0.0130 (16)	−0.024 (2)
C10	0.111 (3)	0.066 (2)	0.066 (2)	0.047 (2)	0.015 (2)	0.0216 (19)
C11	0.0431 (14)	0.0490 (16)	0.0293 (13)	0.0056 (12)	0.0019 (10)	−0.0009 (12)
C12	0.0578 (17)	0.0505 (16)	0.0347 (14)	0.0099 (14)	0.0022 (12)	0.0079 (13)
C13	0.072 (2)	0.0474 (17)	0.0444 (16)	−0.0005 (15)	0.0111 (15)	0.0149 (14)
C14	0.0547 (18)	0.0590 (19)	0.0501 (17)	−0.0141 (15)	0.0158 (14)	0.0024 (15)
C15	0.0344 (14)	0.0686 (19)	0.0420 (15)	0.0026 (13)	0.0103 (12)	−0.0019 (14)
C16	0.0430 (15)	0.0456 (15)	0.0348 (13)	0.0078 (12)	0.0053 (11)	−0.0050 (12)
C17	0.0451 (16)	0.070 (2)	0.0345 (14)	0.0059 (14)	0.0006 (11)	−0.0029 (14)
C18	0.0523 (17)	0.0608 (19)	0.0410 (16)	0.0005 (14)	−0.0067 (13)	−0.0061 (14)
C19	0.0588 (19)	0.071 (2)	0.0386 (16)	0.0002 (16)	−0.0046 (13)	−0.0105 (15)
C20	0.076 (2)	0.090 (3)	0.056 (2)	0.017 (2)	−0.0111 (18)	−0.025 (2)
C21	0.098 (3)	0.064 (2)	0.094 (3)	0.011 (2)	−0.029 (3)	−0.025 (2)
C22	0.085 (3)	0.072 (3)	0.134 (4)	−0.017 (2)	−0.011 (3)	−0.011 (3)
C23	0.062 (2)	0.083 (3)	0.082 (3)	−0.0110 (19)	−0.0005 (19)	−0.010 (2)
C24	0.0333 (12)	0.0270 (11)	0.0369 (13)	0.0009 (9)	0.0030 (10)	−0.0010 (10)
C25	0.0373 (14)	0.0398 (14)	0.0433 (15)	0.0013 (11)	0.0005 (11)	0.0030 (12)
C26	0.0327 (14)	0.0466 (16)	0.075 (2)	0.0001 (12)	0.0008 (13)	0.0017 (15)
C27	0.0414 (15)	0.0422 (15)	0.068 (2)	−0.0007 (12)	0.0202 (14)	0.0022 (14)
C28	0.0553 (17)	0.0404 (15)	0.0463 (16)	0.0010 (12)	0.0190 (13)	0.0019 (13)
C29	0.0401 (14)	0.0387 (14)	0.0372 (14)	−0.0024 (11)	0.0049 (11)	0.0012 (11)
C30	0.0372 (13)	0.0360 (13)	0.0326 (13)	0.0028 (10)	0.0041 (10)	0.0002 (11)
C31	0.0379 (15)	0.0430 (16)	0.072 (2)	0.0001 (12)	−0.0039 (13)	0.0034 (15)
C32	0.0373 (16)	0.066 (2)	0.099 (3)	0.0059 (15)	−0.0105 (17)	−0.002 (2)
C33	0.057 (2)	0.062 (2)	0.073 (2)	0.0272 (17)	−0.0106 (16)	0.0002 (18)
C34	0.067 (2)	0.0384 (15)	0.061 (2)	0.0130 (14)	0.0057 (15)	0.0039 (14)
C35	0.0447 (15)	0.0371 (14)	0.0520 (17)	0.0013 (11)	0.0023 (12)	0.0009 (12)
C36	0.0355 (12)	0.0326 (12)	0.0350 (13)	−0.0090 (10)	0.0059 (10)	0.0001 (10)
C37	0.0435 (15)	0.0517 (16)	0.0434 (15)	−0.0060 (12)	0.0102 (12)	−0.0063 (13)
C38	0.062 (2)	0.079 (2)	0.0492 (18)	−0.0113 (17)	0.0230 (16)	−0.0165 (17)
C39	0.070 (2)	0.096 (3)	0.0317 (15)	−0.026 (2)	0.0119 (15)	−0.0050 (17)
C40	0.0614 (19)	0.068 (2)	0.0391 (15)	−0.0120 (15)	−0.0020 (14)	0.0152 (15)
C41	0.0514 (16)	0.0464 (16)	0.0362 (14)	−0.0020 (12)	0.0033 (12)	0.0040 (12)
C42	0.0266 (11)	0.0339 (12)	0.0362 (13)	0.0017 (9)	−0.0016 (9)	0.0018 (10)
C43	0.0401 (14)	0.0414 (14)	0.0398 (14)	0.0044 (11)	−0.0008 (11)	−0.0036 (12)
C44	0.0418 (15)	0.0509 (17)	0.0588 (19)	0.0074 (13)	−0.0048 (13)	−0.0185 (15)
C45	0.0464 (16)	0.0359 (15)	0.085 (2)	−0.0020 (13)	−0.0142 (16)	−0.0098 (16)
C46	0.0550 (18)	0.0428 (16)	0.069 (2)	−0.0151 (14)	−0.0025 (15)	0.0080 (16)
C47	0.0513 (16)	0.0435 (15)	0.0456 (16)	−0.0110 (12)	0.0045 (12)	0.0013 (13)
B	0.0320 (14)	0.0307 (14)	0.0338 (14)	0.0008 (10)	0.0034 (11)	0.0024 (11)

### Geometric parameters (Å, °)

**Table:**

Ru—C1	2.164 (3)	C16—H16	0.9600
Ru—C2	2.180 (2)	C19—H19	0.9500
Ru—C3	2.191 (3)	C20—H20	0.9500
Ru—C4	2.185 (3)	C21—H21	0.9500
Ru—C5	2.178 (3)	C22—H22	0.9700
Ru—C11	2.230 (3)	C23—H23	0.9500
Ru—C12	2.224 (3)	C24—C25	1.411 (3)
Ru—C13	2.224 (3)	C24—C29	1.404 (4)
Ru—C14	2.204 (3)	C24—B	1.656 (3)
Ru—C15	2.203 (3)	C25—C26	1.396 (4)
Ru—C16	2.210 (3)	C26—C27	1.373 (4)
O1—C17	1.207 (3)	C27—C28	1.369 (4)
C1—C2	1.438 (4)	C28—C29	1.387 (4)
C1—C5	1.423 (5)	C30—C31	1.386 (3)
C1—C6	1.498 (5)	C30—C35	1.401 (4)
C2—C3	1.425 (4)	C30—B	1.650 (3)
C2—C7	1.497 (5)	C31—C32	1.385 (5)
C3—C4	1.420 (4)	C32—C33	1.370 (5)
C3—C8	1.497 (4)	C33—C34	1.366 (5)
C4—C5	1.428 (4)	C34—C35	1.394 (4)
C4—C9	1.502 (4)	C36—C37	1.393 (4)
C5—C10	1.511 (5)	C36—C41	1.406 (4)
C11—C12	1.433 (4)	C36—B	1.651 (4)
C11—C16	1.417 (3)	C37—C38	1.401 (4)
C11—C17	1.512 (4)	C38—C39	1.368 (5)
C12—C13	1.401 (5)	C39—C40	1.370 (5)
C13—C14	1.404 (5)	C40—C41	1.381 (4)
C14—C15	1.415 (5)	C42—C43	1.403 (4)
C15—C16	1.428 (4)	C42—C47	1.399 (4)
C17—C18	1.496 (5)	C42—B	1.662 (4)
C18—C19	1.388 (5)	C43—C44	1.396 (4)
C18—C23	1.386 (5)	C44—C45	1.377 (4)
C19—C20	1.380 (5)	C45—C46	1.372 (4)
C20—C21	1.377 (6)	C46—C47	1.393 (4)
C21—C22	1.350 (7)	C25—H25	0.9400
C22—C23	1.381 (6)	C26—H26	0.9600
C6—H6A	0.9700	C27—H27	0.9500
C6—H6B	0.9500	C28—H28	0.9500
C6—H6C	0.9600	C29—H29	0.9500
C7—H7A	0.9500	C31—H31	0.9600
C7—H7B	0.9600	C32—H32	0.9500
C7—H7C	0.9700	C33—H33	0.9500
C8—H8A	0.9500	C34—H34	0.9500
C8—H8B	0.9700	C35—H35	0.9500
C8—H8C	0.9600	C37—H37	0.9500
C9—H9A	0.9600	C38—H38	0.9400
C9—H9B	0.9600	C39—H39	0.9500
C9—H9C	0.9500	C40—H40	0.9500
C10—H10A	0.9500	C41—H41	0.9500
C10—H10B	0.9700	C43—H43	0.9500
C10—H10C	0.9500	C44—H44	0.9400
C12—H12	0.9600	C45—H45	0.9500
C13—H13	0.9600	C46—H46	0.9400
C14—H14	0.9700	C47—H47	0.9500
C15—H15	0.9600		
			
O1···H6B	2.7600	C27···H13^iii^	2.7500
O1···H12	2.5600	C28···H13^iii^	2.8200
O1···H23	2.5700	C29···H13^iii^	3.0000
C1···C3	2.319 (4)	C29···H35	2.8400
C1···C4	2.303 (4)	C29···H43	2.6800
C1···C7	2.617 (6)	C30···H29	2.7700
C1···C10	2.615 (6)	C30···H37	2.5500
C1···C11	3.563 (4)	C31···H37	2.9900
C2···C4	2.297 (3)	C31···H23^iv^	3.0700
C2···C5	2.311 (4)	C34···H7A^iii^	3.0300
C2···C6	2.623 (5)	C35···H37	2.9100
C2···C8	2.616 (5)	C35···H29	2.9000
C2···C12	3.571 (4)	C36···H47	2.5700
C3···C1	2.319 (4)	C36···H25	2.7500
C3···C5	2.315 (4)	C37···H8A^iii^	2.7800
C3···C7	2.601 (4)	C38···H8A^iii^	2.8000
C3···C9	2.586 (4)	C41···H25	2.5400
C3···C13	3.543 (4)	C41···H47	2.7200
C4···C1	2.303 (4)	C42···H41	3.0100
C4···C2	2.297 (3)	C42···H31	2.6600
C4···C8	2.591 (5)	C42···H15	2.9400
C4···C10	2.621 (5)	C43···H15	2.9500
C4···C14	3.519 (4)	C44···H10B^vi^	2.9300
C4···C15	3.592 (4)	C44···H15	2.9900
C5···C2	2.311 (4)	C45···H15	2.9800
C5···C3	2.315 (4)	C45···H7B^iv^	2.8300
C5···C6	2.599 (6)	C46···H15	2.9400
C5···C9	2.619 (5)	C47···H15	2.9200
C5···C15	3.545 (4)	C47···H41	3.0800
C5···C16	3.556 (4)	C47···H31	2.9600
C7···C45^i^	3.495 (5)	H6A···H10A	2.4200
C8···C37^ii^	3.467 (5)	H6A···C10	2.7900
C10···C28	3.563 (5)	H6B···C17	2.7500
C11···C15	2.463 (3)	H6B···O1	2.7600
C11···C13	2.460 (4)	H6C···H10A^v^	2.5600
C11···C1	3.563 (4)	H6C···C10^v^	3.0700
C11···C14	2.828 (4)	H6C···C7	3.0300
C12···C17	2.516 (4)	H6C···H7B	2.5200
C12···C14	2.428 (5)	H6C···C5^v^	3.0800
C12···C15	2.831 (4)	H7A···C34^ii^	3.0300
C12···C16	2.459 (4)	H7B···C6	2.9700
C12···C2	3.571 (4)	H7B···C45^i^	2.8300
C13···C15	2.454 (4)	H7B···H6C	2.5200
C13···C3	3.543 (4)	H7C···C8	2.8500
C13···C16	2.838 (4)	H7C···H8A	2.4500
C13···C27^ii^	3.504 (4)	H8A···C37^ii^	2.7800
C13···C11	2.460 (4)	H8A···H7C	2.4500
C14···C11	2.828 (4)	H8A···C7	3.0500
C14···C12	2.428 (5)	H8A···C38^ii^	2.8000
C14···C4	3.519 (4)	H8B···H9C	2.1900
C14···C16	2.454 (4)	H8B···C9	2.7100
C15···C4	3.592 (4)	H8C···C25^ii^	2.9400
C15···C11	2.463 (3)	H9B···H10B	2.2500
C15···C5	3.545 (4)	H9B···C10	2.8900
C15···C13	2.454 (4)	H9C···C8	2.8400
C15···C12	2.831 (4)	H9C···H8B	2.1900
C16···C17	2.575 (3)	H10A···C6	3.0300
C16···C19	3.121 (4)	H10A···H6A	2.4200
C16···C12	2.459 (4)	H10A···H6C^v^	2.5600
C16···C13	2.838 (4)	H10B···C44^vi^	2.9300
C16···C5	3.556 (4)	H10B···H44^vi^	2.6000
C16···C14	2.454 (4)	H10B···H9B	2.2500
C19···C16	3.121 (4)	H10B···C9	2.8200
C25···C41	3.229 (4)	H12···H35^ii^	2.6000
C27···C13^iii^	3.504 (4)	H12···C21^viii^	3.0300
C28···C10	3.563 (5)	H12···O1	2.5600
C29···C35	3.204 (4)	H13···C27^ii^	2.7500
C29···C43	3.352 (4)	H13···C28^ii^	2.8200
C31···C37	3.562 (4)	H13···C25^ii^	3.0100
C35···C29	3.204 (4)	H13···C26^ii^	2.8400
C35···C37	3.502 (4)	H13···C29^ii^	3.0000
C37···C35	3.502 (4)	H15···C46	2.9400
C37···C8^iii^	3.467 (5)	H15···C43	2.9500
C37···C31	3.562 (4)	H15···C47	2.9200
C41···C47	3.200 (4)	H15···C42	2.9400
C41···C25	3.229 (4)	H15···C44	2.9900
C43···C29	3.352 (4)	H15···C45	2.9800
C45···C7^iv^	3.495 (5)	H16···C25	2.9900
C47···C41	3.200 (4)	H16···C19	2.6900
C5···H6C^v^	3.0800	H16···C18	2.8500
C6···H7B	2.9700	H16···H19	2.3800
C6···H10A	3.0300	H16···H41	2.6000
C7···H6C	3.0300	H19···C11	2.7500
C7···H8A	3.0500	H19···C16	2.6600
C8···H9C	2.8400	H19···H16	2.3800
C8···H7C	2.8500	H23···O1	2.5700
C9···H8B	2.7100	H23···C31^i^	3.0700
C9···H10B	2.8200	H25···C36	2.7500
C9···H29^vi^	3.0800	H25···C41	2.5400
C10···H9B	2.8900	H25···H41	2.2300
C10···H6C^v^	3.0700	H29···C30	2.7700
C10···H6A	2.7900	H29···C35	2.9000
C11···H19	2.7500	H29···H43	2.4100
C11···H16	2.0700	H29···C9^vi^	3.0800
C11···H12	2.0800	H31···C42	2.6600
C12···H13	2.0600	H31···C47	2.9600
C13···H12	2.0600	H34···H46^ix^	2.4600
C13···H14	2.0500	H35···C24	2.7300
C14···H39^vii^	2.9300	H35···C29	2.8400
C14···H13	2.0600	H35···H12^iii^	2.6000
C14···H15	2.0800	H37···C30	2.5500
C15···H16	2.0800	H37···C31	2.9900
C15···H14	2.0800	H37···C35	2.9100
C15···H39^vii^	2.9700	H39···C14^vii^	2.9300
C16···H19	2.6600	H39···C15^vii^	2.9700
C16···H15	2.0800	H41···C25	2.9000
C17···H6B	2.7500	H41···C42	3.0100
C18···H16	2.8500	H41···C47	3.0800
C19···H16	2.6900	H41···H16	2.6000
C21···H12^viii^	3.0300	H41···H25	2.2300
C24···H35	2.7300	H43···C24	2.8500
C24···H43	2.8500	H43···C29	2.6800
C25···H8C^iii^	2.9400	H43···H29	2.4100
C25···H13^iii^	3.0100	H44···H10B^vi^	2.6000
C25···H41	2.9000	H46···H34^x^	2.4600
C25···H16	2.9900	H47···C36	2.5700
C26···H13^iii^	2.8400	H47···C41	2.7200
			
C1—Ru—C2	38.67 (12)	C2—C7—H7C	110.00
C1—Ru—C3	64.34 (10)	H7A—C7—H7B	109.00
C1—Ru—C4	63.96 (11)	H7A—C7—H7C	108.00
C1—Ru—C5	38.25 (13)	H7B—C7—H7C	107.00
C1—Ru—C11	108.37 (10)	C3—C8—H8A	112.00
C1—Ru—C12	125.61 (12)	C3—C8—H8B	110.00
C1—Ru—C13	155.02 (13)	C3—C8—H8C	111.00
C1—Ru—C14	167.46 (12)	H8A—C8—H8B	108.00
C1—Ru—C15	135.40 (12)	H8A—C8—H8C	109.00
C1—Ru—C16	112.23 (10)	H8B—C8—H8C	107.00
C2—Ru—C3	38.06 (10)	C4—C9—H9A	110.00
C2—Ru—C4	63.49 (9)	C4—C9—H9B	111.00
C2—Ru—C5	64.03 (11)	C4—C9—H9C	111.00
C2—Ru—C11	117.84 (9)	H9A—C9—H9B	108.00
C2—Ru—C12	108.37 (11)	H9A—C9—H9C	108.00
C2—Ru—C13	119.78 (12)	H9B—C9—H9C	109.00
C2—Ru—C14	146.56 (12)	C5—C10—H10A	111.00
C2—Ru—C15	172.00 (10)	C5—C10—H10B	110.00
C2—Ru—C16	143.45 (10)	C5—C10—H10C	111.00
C3—Ru—C4	37.87 (10)	H10A—C10—H10B	108.00
C3—Ru—C5	63.99 (10)	H10A—C10—H10C	109.00
C3—Ru—C11	150.40 (9)	H10B—C10—H10C	108.00
C3—Ru—C12	121.10 (11)	Ru—C12—H12	129.00
C3—Ru—C13	106.72 (11)	C11—C12—H12	120.00
C3—Ru—C14	113.97 (11)	C13—C12—H12	120.00
C3—Ru—C15	138.96 (10)	Ru—C13—H13	130.00
C3—Ru—C16	171.48 (10)	C12—C13—H13	120.00
C4—Ru—C5	38.22 (11)	C14—C13—H13	120.00
C4—Ru—C11	167.09 (11)	Ru—C14—H14	130.00
C4—Ru—C12	155.35 (11)	C13—C14—H14	119.00
C4—Ru—C13	124.47 (11)	C15—C14—H14	120.00
C4—Ru—C14	106.59 (11)	Ru—C15—H15	129.00
C4—Ru—C15	109.90 (9)	C14—C15—H15	121.00
C4—Ru—C16	133.76 (10)	C16—C15—H15	120.00
C5—Ru—C11	129.39 (11)	Ru—C16—H16	129.00
C5—Ru—C12	162.06 (12)	C11—C16—H16	120.00
C5—Ru—C13	161.18 (12)	C15—C16—H16	120.00
C5—Ru—C14	129.25 (13)	C18—C19—H19	120.00
C5—Ru—C15	108.03 (12)	C20—C19—H19	120.00
C5—Ru—C16	108.26 (10)	C19—C20—H20	120.00
C11—Ru—C12	37.53 (11)	C21—C20—H20	120.00
C11—Ru—C13	67.07 (11)	C20—C21—H21	119.00
C11—Ru—C14	79.23 (11)	C22—C21—H21	121.00
C11—Ru—C15	67.49 (9)	C21—C22—H22	120.00
C11—Ru—C16	37.21 (9)	C23—C22—H22	119.00
C12—Ru—C13	36.73 (12)	C18—C23—H23	120.00
C12—Ru—C14	66.49 (12)	C22—C23—H23	120.00
C12—Ru—C15	79.52 (11)	C25—C24—C29	114.5 (2)
C12—Ru—C16	67.38 (11)	C25—C24—B	124.2 (2)
C13—Ru—C14	36.96 (13)	C29—C24—B	121.4 (2)
C13—Ru—C15	67.32 (12)	C24—C25—C26	121.9 (3)
C13—Ru—C16	79.60 (11)	C25—C26—C27	120.8 (2)
C14—Ru—C15	37.46 (12)	C26—C27—C28	119.3 (2)
C14—Ru—C16	67.53 (11)	C27—C28—C29	120.0 (3)
C15—Ru—C16	37.76 (10)	C24—C29—C28	123.5 (2)
Ru—C1—C2	71.27 (16)	C31—C30—C35	114.6 (2)
Ru—C1—C5	71.42 (17)	C31—C30—B	123.2 (2)
Ru—C1—C6	124.4 (2)	C35—C30—B	122.0 (2)
C2—C1—C5	107.7 (3)	C30—C31—C32	122.7 (3)
C2—C1—C6	126.6 (3)	C31—C32—C33	121.0 (3)
C5—C1—C6	125.7 (3)	C32—C33—C34	118.7 (3)
Ru—C2—C1	70.06 (16)	C33—C34—C35	120.0 (3)
Ru—C2—C3	71.40 (14)	C30—C35—C34	123.1 (2)
Ru—C2—C7	126.7 (2)	C37—C36—C41	114.3 (2)
C1—C2—C3	108.1 (2)	C37—C36—B	123.5 (2)
C1—C2—C7	126.1 (3)	C41—C36—B	122.0 (2)
C3—C2—C7	125.7 (3)	C36—C37—C38	122.2 (3)
Ru—C3—C2	70.55 (15)	C37—C38—C39	120.6 (3)
Ru—C3—C4	70.82 (15)	C38—C39—C40	119.3 (3)
Ru—C3—C8	126.0 (2)	C39—C40—C41	119.5 (3)
C2—C3—C4	107.6 (2)	C36—C41—C40	123.9 (3)
C2—C3—C8	127.1 (3)	C43—C42—C47	114.5 (2)
C4—C3—C8	125.3 (3)	C43—C42—B	123.0 (2)
Ru—C4—C3	71.31 (15)	C47—C42—B	122.4 (2)
Ru—C4—C5	70.64 (15)	C42—C43—C44	122.9 (2)
Ru—C4—C9	126.30 (19)	C43—C44—C45	120.1 (3)
C3—C4—C5	108.7 (2)	C44—C45—C46	119.0 (3)
C3—C4—C9	124.5 (3)	C45—C46—C47	120.4 (3)
C5—C4—C9	126.7 (3)	C42—C47—C46	123.0 (3)
Ru—C5—C1	70.33 (16)	C26—C25—H25	119.00
Ru—C5—C4	71.14 (15)	C24—C25—H25	119.00
Ru—C5—C10	125.5 (2)	C25—C26—H26	119.00
C1—C5—C4	107.8 (3)	C27—C26—H26	120.00
C1—C5—C10	126.0 (3)	C28—C27—H27	121.00
C4—C5—C10	126.2 (3)	C26—C27—H27	120.00
Ru—C11—C12	71.00 (16)	C27—C28—H28	120.00
Ru—C11—C16	70.62 (15)	C29—C28—H28	120.00
Ru—C11—C17	126.44 (17)	C24—C29—H29	118.00
C12—C11—C16	119.3 (2)	C28—C29—H29	118.00
C12—C11—C17	117.4 (2)	C32—C31—H31	119.00
C16—C11—C17	123.1 (3)	C30—C31—H31	118.00
Ru—C12—C11	71.47 (16)	C31—C32—H32	120.00
Ru—C12—C13	71.64 (18)	C33—C32—H32	119.00
C11—C12—C13	120.5 (3)	C32—C33—H33	121.00
Ru—C13—C12	71.63 (18)	C34—C33—H33	121.00
Ru—C13—C14	70.76 (18)	C35—C34—H34	120.00
C12—C13—C14	119.9 (3)	C33—C34—H34	120.00
Ru—C14—C13	72.28 (19)	C30—C35—H35	119.00
Ru—C14—C15	71.22 (17)	C34—C35—H35	118.00
C13—C14—C15	121.0 (3)	C36—C37—H37	119.00
Ru—C15—C14	71.33 (18)	C38—C37—H37	119.00
Ru—C15—C16	71.39 (14)	C39—C38—H38	120.00
C14—C15—C16	119.3 (2)	C37—C38—H38	120.00
Ru—C16—C11	72.17 (15)	C38—C39—H39	120.00
Ru—C16—C15	70.86 (15)	C40—C39—H39	121.00
C11—C16—C15	119.9 (2)	C41—C40—H40	121.00
O1—C17—C11	118.7 (3)	C39—C40—H40	120.00
O1—C17—C18	121.2 (3)	C36—C41—H41	118.00
C11—C17—C18	120.0 (2)	C40—C41—H41	118.00
C17—C18—C19	122.6 (3)	C42—C43—H43	119.00
C17—C18—C23	118.5 (3)	C44—C43—H43	118.00
C19—C18—C23	118.9 (3)	C45—C44—H44	120.00
C18—C19—C20	120.1 (3)	C43—C44—H44	120.00
C19—C20—C21	120.1 (3)	C44—C45—H45	120.00
C20—C21—C22	119.9 (4)	C46—C45—H45	121.00
C21—C22—C23	121.2 (4)	C45—C46—H46	119.00
C18—C23—C22	119.8 (4)	C47—C46—H46	121.00
C1—C6—H6A	110.00	C46—C47—H47	118.00
C1—C6—H6B	112.00	C42—C47—H47	119.00
C1—C6—H6C	111.00	C24—B—C36	112.32 (19)
H6A—C6—H6B	108.00	C24—B—C42	109.78 (18)
H6A—C6—H6C	107.00	C30—B—C42	109.23 (18)
H6B—C6—H6C	108.00	C36—B—C42	107.80 (19)
C2—C7—H7A	112.00	C30—B—C36	108.76 (19)
C2—C7—H7B	111.00	C24—B—C30	108.91 (19)
			
C2—Ru—C1—C5	116.9 (2)	C3—Ru—C14—C15	−140.99 (16)
C2—Ru—C1—C6	−122.0 (4)	C4—Ru—C14—C13	125.88 (18)
C3—Ru—C1—C2	−37.10 (15)	C4—Ru—C14—C15	−101.24 (18)
C3—Ru—C1—C5	79.84 (17)	C5—Ru—C14—C13	161.28 (17)
C3—Ru—C1—C6	−159.1 (3)	C5—Ru—C14—C15	−65.9 (2)
C4—Ru—C1—C2	−79.37 (17)	C11—Ru—C14—C13	−65.99 (18)
C4—Ru—C1—C5	37.57 (16)	C11—Ru—C14—C15	66.89 (17)
C4—Ru—C1—C6	158.6 (3)	C12—Ru—C14—C13	−28.89 (18)
C5—Ru—C1—C2	−116.9 (2)	C12—Ru—C14—C15	103.99 (19)
C5—Ru—C1—C6	121.0 (4)	C13—Ru—C14—C15	132.9 (3)
C11—Ru—C1—C2	111.87 (16)	C15—Ru—C14—C13	−132.9 (3)
C11—Ru—C1—C5	−131.20 (16)	C16—Ru—C14—C13	−102.99 (19)
C11—Ru—C1—C6	−10.2 (3)	C16—Ru—C14—C15	29.89 (16)
C12—Ru—C1—C2	74.06 (19)	C1—Ru—C15—C14	164.21 (18)
C12—Ru—C1—C5	−169.01 (16)	C1—Ru—C15—C16	−64.6 (2)
C12—Ru—C1—C6	−48.0 (3)	C3—Ru—C15—C14	61.2 (2)
C13—Ru—C1—C2	36.9 (3)	C3—Ru—C15—C16	−167.59 (16)
C13—Ru—C1—C5	153.8 (2)	C4—Ru—C15—C14	91.54 (19)
C13—Ru—C1—C6	−85.1 (4)	C4—Ru—C15—C16	−137.22 (16)
C15—Ru—C1—C2	−171.89 (15)	C5—Ru—C15—C14	132.00 (18)
C15—Ru—C1—C5	−55.0 (2)	C5—Ru—C15—C16	−96.77 (17)
C15—Ru—C1—C6	66.1 (3)	C11—Ru—C15—C14	−102.02 (19)
C16—Ru—C1—C2	151.44 (15)	C11—Ru—C15—C16	29.21 (15)
C16—Ru—C1—C5	−91.63 (17)	C12—Ru—C15—C14	−64.81 (18)
C16—Ru—C1—C6	29.4 (3)	C12—Ru—C15—C16	66.42 (17)
C1—Ru—C2—C3	−118.1 (2)	C13—Ru—C15—C14	−28.52 (18)
C1—Ru—C2—C7	120.7 (4)	C13—Ru—C15—C16	102.71 (18)
C3—Ru—C2—C1	118.1 (2)	C14—Ru—C15—C16	131.2 (2)
C3—Ru—C2—C7	−121.2 (4)	C16—Ru—C15—C14	−131.2 (2)
C4—Ru—C2—C1	80.68 (18)	C1—Ru—C16—C11	−91.43 (17)
C4—Ru—C2—C3	−37.45 (16)	C1—Ru—C16—C15	136.77 (17)
C4—Ru—C2—C7	−158.6 (4)	C2—Ru—C16—C11	−61.3 (2)
C5—Ru—C2—C1	37.88 (17)	C2—Ru—C16—C15	166.88 (18)
C5—Ru—C2—C3	−80.26 (17)	C4—Ru—C16—C11	−166.04 (16)
C5—Ru—C2—C7	158.6 (4)	C4—Ru—C16—C15	62.2 (2)
C11—Ru—C2—C1	−84.87 (18)	C5—Ru—C16—C11	−132.10 (17)
C11—Ru—C2—C3	156.99 (15)	C5—Ru—C16—C15	96.10 (18)
C11—Ru—C2—C7	35.8 (4)	C11—Ru—C16—C15	−131.8 (2)
C12—Ru—C2—C1	−124.55 (17)	C12—Ru—C16—C11	29.32 (16)
C12—Ru—C2—C3	117.32 (16)	C12—Ru—C16—C15	−102.49 (19)
C12—Ru—C2—C7	−3.9 (4)	C13—Ru—C16—C11	65.58 (17)
C13—Ru—C2—C1	−163.01 (17)	C13—Ru—C16—C15	−66.22 (18)
C13—Ru—C2—C3	78.85 (18)	C14—Ru—C16—C11	102.14 (18)
C13—Ru—C2—C7	−42.3 (4)	C14—Ru—C16—C15	−29.67 (17)
C14—Ru—C2—C1	160.5 (2)	C15—Ru—C16—C11	131.8 (2)
C14—Ru—C2—C3	42.4 (3)	Ru—C1—C2—C3	61.58 (18)
C14—Ru—C2—C7	−78.8 (4)	Ru—C1—C2—C7	−121.4 (3)
C16—Ru—C2—C1	−48.0 (2)	C5—C1—C2—Ru	−62.5 (2)
C16—Ru—C2—C3	−166.14 (16)	C5—C1—C2—C3	−0.9 (3)
C16—Ru—C2—C7	72.7 (4)	C5—C1—C2—C7	176.1 (3)
C1—Ru—C3—C2	37.69 (17)	C6—C1—C2—Ru	119.4 (3)
C1—Ru—C3—C4	−79.89 (18)	C6—C1—C2—C3	−179.0 (3)
C1—Ru—C3—C8	159.9 (3)	C6—C1—C2—C7	−2.1 (5)
C2—Ru—C3—C4	−117.6 (2)	Ru—C1—C5—C4	−61.64 (19)
C2—Ru—C3—C8	122.2 (3)	Ru—C1—C5—C10	120.1 (3)
C4—Ru—C3—C2	117.6 (2)	C2—C1—C5—Ru	62.42 (19)
C4—Ru—C3—C8	−120.2 (3)	C2—C1—C5—C4	0.8 (3)
C5—Ru—C3—C2	80.38 (18)	C2—C1—C5—C10	−177.5 (3)
C5—Ru—C3—C4	−37.19 (17)	C6—C1—C5—Ru	−119.5 (3)
C5—Ru—C3—C8	−157.4 (3)	C6—C1—C5—C4	178.9 (3)
C11—Ru—C3—C2	−44.4 (3)	C6—C1—C5—C10	0.7 (5)
C11—Ru—C3—C4	−161.97 (19)	Ru—C2—C3—C4	61.46 (18)
C11—Ru—C3—C8	77.8 (3)	Ru—C2—C3—C8	−120.9 (3)
C12—Ru—C3—C2	−80.00 (19)	C1—C2—C3—Ru	−60.73 (18)
C12—Ru—C3—C4	162.43 (16)	C1—C2—C3—C4	0.7 (3)
C12—Ru—C3—C8	42.2 (3)	C1—C2—C3—C8	178.3 (3)
C13—Ru—C3—C2	−117.23 (17)	C7—C2—C3—Ru	122.3 (3)
C13—Ru—C3—C4	125.19 (17)	C7—C2—C3—C4	−176.3 (3)
C13—Ru—C3—C8	5.0 (3)	C7—C2—C3—C8	1.3 (5)
C14—Ru—C3—C2	−156.01 (17)	Ru—C3—C4—C5	61.03 (19)
C14—Ru—C3—C4	86.42 (18)	Ru—C3—C4—C9	−121.7 (3)
C14—Ru—C3—C8	−33.8 (3)	C2—C3—C4—Ru	−61.28 (18)
C15—Ru—C3—C2	168.32 (17)	C2—C3—C4—C5	−0.3 (3)
C15—Ru—C3—C4	50.7 (2)	C2—C3—C4—C9	177.0 (3)
C15—Ru—C3—C8	−69.5 (3)	C8—C3—C4—Ru	121.1 (3)
C1—Ru—C4—C3	80.98 (17)	C8—C3—C4—C5	−177.9 (3)
C1—Ru—C4—C5	−37.60 (18)	C8—C3—C4—C9	−0.7 (5)
C1—Ru—C4—C9	−159.5 (3)	Ru—C4—C5—C1	61.11 (19)
C2—Ru—C4—C3	37.64 (15)	Ru—C4—C5—C10	−120.7 (3)
C2—Ru—C4—C5	−80.94 (19)	C3—C4—C5—Ru	−61.45 (19)
C2—Ru—C4—C9	157.2 (3)	C3—C4—C5—C1	−0.3 (3)
C3—Ru—C4—C5	−118.6 (2)	C3—C4—C5—C10	177.9 (3)
C3—Ru—C4—C9	119.6 (3)	C9—C4—C5—Ru	121.4 (3)
C5—Ru—C4—C3	118.6 (2)	C9—C4—C5—C1	−177.5 (3)
C5—Ru—C4—C9	−121.9 (3)	C9—C4—C5—C10	0.7 (5)
C12—Ru—C4—C3	−38.3 (3)	Ru—C11—C12—C13	−54.4 (2)
C12—Ru—C4—C5	−156.9 (3)	C16—C11—C12—Ru	53.4 (2)
C12—Ru—C4—C9	81.3 (4)	C16—C11—C12—C13	−0.9 (4)
C13—Ru—C4—C3	−71.68 (19)	C17—C11—C12—Ru	−121.9 (2)
C13—Ru—C4—C5	169.74 (19)	C17—C11—C12—C13	−176.3 (3)
C13—Ru—C4—C9	47.9 (3)	Ru—C11—C16—C15	54.4 (2)
C14—Ru—C4—C3	−107.89 (16)	C12—C11—C16—Ru	−53.6 (2)
C14—Ru—C4—C5	133.53 (19)	C12—C11—C16—C15	0.8 (4)
C14—Ru—C4—C9	11.7 (3)	C17—C11—C16—Ru	121.5 (2)
C15—Ru—C4—C3	−147.27 (16)	C17—C11—C16—C15	175.8 (2)
C15—Ru—C4—C5	94.15 (19)	Ru—C11—C17—O1	−58.8 (4)
C15—Ru—C4—C9	−27.7 (3)	Ru—C11—C17—C18	122.0 (2)
C16—Ru—C4—C3	177.58 (14)	C12—C11—C17—O1	27.0 (4)
C16—Ru—C4—C5	59.0 (2)	C12—C11—C17—C18	−152.2 (3)
C16—Ru—C4—C9	−62.9 (3)	C16—C11—C17—O1	−148.1 (3)
C1—Ru—C5—C4	117.7 (2)	C16—C11—C17—C18	32.6 (4)
C1—Ru—C5—C10	−120.8 (4)	Ru—C12—C13—C14	−53.8 (3)
C2—Ru—C5—C1	−38.29 (17)	C11—C12—C13—Ru	54.3 (2)
C2—Ru—C5—C4	79.40 (17)	C11—C12—C13—C14	0.5 (4)
C2—Ru—C5—C10	−159.1 (3)	Ru—C13—C14—C15	−54.1 (3)
C3—Ru—C5—C1	−80.83 (18)	C12—C13—C14—Ru	54.2 (3)
C3—Ru—C5—C4	36.86 (15)	C12—C13—C14—C15	0.1 (5)
C3—Ru—C5—C10	158.4 (3)	Ru—C14—C15—C16	−54.8 (2)
C4—Ru—C5—C1	−117.7 (2)	C13—C14—C15—Ru	54.6 (3)
C4—Ru—C5—C10	121.5 (4)	C13—C14—C15—C16	−0.3 (4)
C11—Ru—C5—C1	67.5 (2)	Ru—C15—C16—C11	−55.0 (2)
C11—Ru—C5—C4	−174.81 (14)	C14—C15—C16—Ru	54.8 (2)
C11—Ru—C5—C10	−53.3 (3)	C14—C15—C16—C11	−0.2 (4)
C14—Ru—C5—C1	178.51 (17)	O1—C17—C18—C19	−153.1 (3)
C14—Ru—C5—C4	−63.8 (2)	O1—C17—C18—C23	24.9 (5)
C14—Ru—C5—C10	57.7 (3)	C11—C17—C18—C19	26.2 (4)
C15—Ru—C5—C1	142.81 (17)	C11—C17—C18—C23	−155.9 (3)
C15—Ru—C5—C4	−99.51 (17)	C17—C18—C19—C20	178.9 (3)
C15—Ru—C5—C10	22.0 (3)	C23—C18—C19—C20	0.9 (5)
C16—Ru—C5—C1	103.00 (17)	C17—C18—C23—C22	−178.7 (4)
C16—Ru—C5—C4	−139.32 (15)	C19—C18—C23—C22	−0.6 (6)
C16—Ru—C5—C10	−17.8 (3)	C18—C19—C20—C21	−0.8 (5)
C1—Ru—C11—C12	−125.09 (19)	C19—C20—C21—C22	0.3 (6)
C1—Ru—C11—C16	102.81 (17)	C20—C21—C22—C23	0.1 (8)
C1—Ru—C11—C17	−14.6 (3)	C21—C22—C23—C18	0.1 (8)
C2—Ru—C11—C12	−84.1 (2)	C29—C24—C25—C26	−2.0 (4)
C2—Ru—C11—C16	143.79 (16)	B—C24—C25—C26	176.9 (2)
C2—Ru—C11—C17	26.4 (3)	C25—C24—C29—C28	1.5 (4)
C3—Ru—C11—C12	−54.9 (3)	B—C24—C29—C28	−177.4 (2)
C3—Ru—C11—C16	172.98 (18)	C25—C24—B—C30	139.7 (2)
C3—Ru—C11—C17	55.6 (3)	C25—C24—B—C36	19.2 (3)
C5—Ru—C11—C12	−162.16 (19)	C25—C24—B—C42	−100.7 (3)
C5—Ru—C11—C16	65.7 (2)	C29—C24—B—C30	−41.5 (3)
C5—Ru—C11—C17	−51.6 (3)	C29—C24—B—C36	−162.0 (2)
C12—Ru—C11—C16	−132.1 (2)	C29—C24—B—C42	78.1 (3)
C12—Ru—C11—C17	110.5 (3)	C24—C25—C26—C27	1.3 (4)
C13—Ru—C11—C12	28.62 (19)	C25—C26—C27—C28	0.0 (4)
C13—Ru—C11—C16	−103.48 (18)	C26—C27—C28—C29	−0.5 (4)
C13—Ru—C11—C17	139.2 (3)	C27—C28—C29—C24	−0.3 (4)
C14—Ru—C11—C12	65.23 (19)	C35—C30—C31—C32	−0.8 (4)
C14—Ru—C11—C16	−66.88 (17)	B—C30—C31—C32	173.7 (3)
C14—Ru—C11—C17	175.8 (3)	C31—C30—C35—C34	0.7 (4)
C15—Ru—C11—C12	102.49 (19)	B—C30—C35—C34	−173.9 (3)
C15—Ru—C11—C16	−29.61 (16)	C31—C30—B—C24	148.1 (2)
C15—Ru—C11—C17	−147.0 (3)	C31—C30—B—C36	−89.2 (3)
C16—Ru—C11—C12	132.1 (2)	C31—C30—B—C42	28.3 (3)
C16—Ru—C11—C17	−117.4 (3)	C35—C30—B—C24	−37.8 (3)
C1—Ru—C12—C11	72.8 (2)	C35—C30—B—C36	84.9 (3)
C1—Ru—C12—C13	−154.75 (19)	C35—C30—B—C42	−157.7 (2)
C2—Ru—C12—C11	112.06 (17)	C30—C31—C32—C33	0.8 (6)
C2—Ru—C12—C13	−115.5 (2)	C31—C32—C33—C34	−0.5 (6)
C3—Ru—C12—C11	151.83 (14)	C32—C33—C34—C35	0.4 (5)
C3—Ru—C12—C13	−75.7 (2)	C33—C34—C35—C30	−0.5 (5)
C4—Ru—C12—C11	178.2 (2)	C41—C36—C37—C38	0.8 (4)
C4—Ru—C12—C13	−49.3 (4)	B—C36—C37—C38	176.4 (3)
C11—Ru—C12—C13	132.5 (3)	C37—C36—C41—C40	−1.9 (4)
C13—Ru—C12—C11	−132.5 (3)	B—C36—C41—C40	−177.6 (3)
C14—Ru—C12—C11	−103.41 (19)	C37—C36—B—C24	121.1 (2)
C14—Ru—C12—C13	29.06 (19)	C37—C36—B—C30	0.4 (3)
C15—Ru—C12—C11	−66.53 (17)	C37—C36—B—C42	−117.9 (2)
C15—Ru—C12—C13	65.9 (2)	C41—C36—B—C24	−63.6 (3)
C16—Ru—C12—C11	−29.08 (15)	C41—C36—B—C30	175.8 (2)
C16—Ru—C12—C13	103.4 (2)	C41—C36—B—C42	57.4 (3)
C1—Ru—C13—C12	55.2 (3)	C36—C37—C38—C39	1.1 (5)
C1—Ru—C13—C14	−172.6 (2)	C37—C38—C39—C40	−1.9 (6)
C2—Ru—C13—C12	80.8 (2)	C38—C39—C40—C41	0.9 (5)
C2—Ru—C13—C14	−147.00 (18)	C39—C40—C41—C36	1.2 (5)
C3—Ru—C13—C12	119.96 (18)	C47—C42—C43—C44	−1.6 (3)
C3—Ru—C13—C14	−107.83 (18)	B—C42—C43—C44	−178.5 (2)
C4—Ru—C13—C12	157.45 (17)	C43—C42—C47—C46	2.4 (4)
C4—Ru—C13—C14	−70.4 (2)	B—C42—C47—C46	179.3 (2)
C11—Ru—C13—C12	−29.21 (18)	C43—C42—B—C24	−41.0 (3)
C11—Ru—C13—C14	103.0 (2)	C43—C42—B—C30	78.3 (3)
C12—Ru—C13—C14	132.2 (3)	C43—C42—B—C36	−163.7 (2)
C14—Ru—C13—C12	−132.2 (3)	C47—C42—B—C24	142.3 (2)
C15—Ru—C13—C12	−103.3 (2)	C47—C42—B—C30	−98.4 (3)
C15—Ru—C13—C14	28.89 (18)	C47—C42—B—C36	19.6 (3)
C16—Ru—C13—C12	−65.92 (19)	C42—C43—C44—C45	−0.4 (4)
C16—Ru—C13—C14	66.28 (18)	C43—C44—C45—C46	1.7 (4)
C2—Ru—C14—C13	59.1 (3)	C44—C45—C46—C47	−1.0 (5)
C2—Ru—C14—C15	−168.04 (17)	C45—C46—C47—C42	−1.2 (5)
C3—Ru—C14—C13	86.14 (19)		

Symmetry codes: (i) *x*+1, *y*, *z*; (ii) −*x*+3/2, *y*−1/2, −*z*+1/2; (iii) −*x*+3/2, *y*+1/2, −*z*+1/2; (iv) *x*−1, *y*, *z*; (v) −*x*+2, −*y*, −*z*; (vi) −*x*+1, −*y*, −*z*; (vii) −*x*+1, −*y*, −*z*+1; (viii) −*x*+2, −*y*, −*z*+1; (ix) −*x*+1/2, *y*+1/2, −*z*+1/2; (x) −*x*+1/2, *y*−1/2, −*z*+1/2.
